# Supplementary material for: AliMarko: A Pipeline for Virus Identification Using an Expert-Guided Approach
Source: Viruses. 2025 Feb 28;17(3):355. doi: 10.3390/v17030355 (PMC11946232; doi:10.3390/v17030355)
Supplement: Supplementary file 1 [file viruses-17-00355-s001.zip › HTML S1. Simulated Sample.html]

 

Simulated Sample


# Sample: simulated sample

## Mapping Summary

| Species | Host source | Coverage width | Mean depth | Genus | Family | Realm |
| --- | --- | --- | --- | --- | --- | --- |
| Miniopterus bat coronavirus 1 | vertebrates | 1.0 | 19.91 | Alphacoronavirus | Coronaviridae | Riboviria |
| Lophivirus Xv2 | bacteria | 0.99 | 19.56 | Lophivirus | Inoviridae | Monodnaviria |
| Murine leukemia virus | vertebrates | 0.24 | 6.57 | Gammaretrovirus | Retroviridae | Riboviria |

## HMM Hit Summary

| HMM | Taxon | Name | Putative Protein | Score |
| --- | --- | --- | --- | --- |
| vHMM\_8398 | Coronaviridae | NODE\_1\_length\_28274\_cov\_12.656579 | spike;glycoprotein | 1568 |
| vHMM\_8393 | Coronaviridae | NODE\_1\_length\_28274\_cov\_12.656579 | replicase;polyprotein;1ab | 6126 |
| vHMM\_8393 | Coronaviridae | NODE\_1\_length\_28274\_cov\_12.656579 | replicase;polyprotein;1ab | 4939 |

---

## Mapping Details

## Host: bacteria

### Lophivirus Xv2

| Fragment | Len | Coverage width | Nucleotide similarity | Mean Depth | MeanMAPQ | SNP Count |
| --- | --- | --- | --- | --- | --- | --- |
| MH206183 | 6564 | 0.9906 | 1.0 | 19.5612 | 0.593 | 0.0 |

## Host: vertebrates

### Miniopterus bat coronavirus 1

| Fragment | Len | Coverage width | Nucleotide similarity | Mean Depth | MeanMAPQ | SNP Count |
| --- | --- | --- | --- | --- | --- | --- |
| EU420138 | 28326 | 0.9982 | 1.0 | 19.911 | 0.6 | 0.0 |

### Murine leukemia virus Sequences of this virus have association with Nextera.

| Fragment | Len | Coverage width | Nucleotide similarity | Mean Depth | MeanMAPQ | SNP Count |
| --- | --- | --- | --- | --- | --- | --- |
| AF033811 | 8332 | 0.2439 | 1.0 | 6.5653 | 0.586 | 3.0 |

---

## HMM Module Results by Contig

## Contigs

### NODE\_1\_length\_28274\_cov\_12.656579:Coronaviridae

### 

| HMM | Taxon | Name | Putative Protein | Score | Score Ratio |
| --- | --- | --- | --- | --- | --- |
| vHMM\_8398 | Coronaviridae | NODE\_1\_length\_28274\_cov\_12.656579 | spike;glycoprotein | 1568 | 106 |
| vHMM\_8393 | Coronaviridae | NODE\_1\_length\_28274\_cov\_12.656579 | replicase;polyprotein;1ab | 6126 | 84.7 |
| vHMM\_8393 | Coronaviridae | NODE\_1\_length\_28274\_cov\_12.656579 | replicase;polyprotein;1ab | 4939 | 68.3 |

#### The best blastn hit: EU420138.1 Miniopterus bat coronavirus 1, complete genome. Alignment length: 28274 bp, identity: 100.0%

### Phylogenetic Tree of vHMM\_8393-Matched Amino Acid Sequences

### Putative protein: replicase;polyprotein;1ab

### Phylogenetic Tree of vHMM\_8398-Matched Amino Acid Sequences

### Putative protein: spike;glycoprotein

### Phylogenetic Tree of vHMM\_8393-Matched Amino Acid Sequences

### Putative protein: replicase;polyprotein;1ab
